# Exosomal miR-409-3p secreted from activated mast cells promotes microglial migration, activation and neuroinflammation by targeting Nr4a2 to activate the NF-κB pathway

**DOI:** 10.1186/s12974-021-02110-5

**Published:** 2021-03-09

**Authors:** Liuqing Hu, Linjie Si, Xiaonan Dai, Hongquan Dong, Zijian Ma, Zhaochu Sun, Nana Li, Huanhuan Sha, Yinan Chen, Yanning Qian, Zhiyuan Zhang

**Affiliations:** 1 Department of Anesthesiology, the First Affiliated Hospital with Nanjing Medical University, Nanjing, 210029 China; 2 Department of Cardiovascular Surgery, the First Affiliated Hospital with Nanjing Medical University, Nanjing, 210029 China; 3 Department of Obstetrics, Obstetrics and Gynecology Hospital Affiliated to Nanjing Medical University, Nanjing, 210004 China; 4 Department of Thoracic Surgery, the First Affiliated Hospital with Nanjing Medical University, Nanjing, 210029 China; 5 Department of Pathology, Nanjing Medical University, Nanjing, 210029 China

**Keywords:** Microglia, Neuroinflammation, Exosome, miR-409-3p

## Abstract

**Objective:**

Neuroinflammation plays a critical role in central nervous system diseases. Exosomal miRNAs released from various cells are implicated in cell-to-cell communication. Prior studies have placed substantial emphasis on the role of cytokines in mast cell-microglia interactions during neuroinflammation. However, it has never been clearly determined whether exosomal miRNAs participate in the interaction between mast cells and microglia and thus mediate neuroinflammation.

**Methods:**

The characteristics of exosomes isolated from cell culture supernatants were confirmed by transmission electron microscopy (TEM), nanoparticle-tracking analysis (NTA) and Western blot. The transfer of PKH67-labelled exosomes and Cy3-labelled miR-409-3p was observed by fluorescence microscopy. Migration and activation of murine BV-2 microglial cells were evaluated through Transwell assays and immunofluorescence staining for Iba1 and CD68. CD86, IL-1β, IL-6 and TNF-α were assessed via qRT-PCR and ELISA. MiR-409-3p was detected by qRT-PCR. Nr4a2 and NF-κB levels were measured by western blot. Regulatory effects were identified by luciferase reporter assays.

**Results:**

Lipopolysaccharide (LPS)-stimulated murine P815 mast cells secreted exosomes that were efficiently taken up by murine BV-2 cells, which promoted murine BV-2 cell migration and activation. LPS-P815 exosomes increased the CD86, IL-1β, IL-6 and TNF-α levels in murine BV-2 microglia. Furthermore, activated mast cells delivered exosomal miR-409-3p to murine BV-2 microglia. Upregulated miR-409-3p promoted murine BV-2 microglial migration, activation and neuroinflammation by targeting Nr4a2 to activate the NF-κB pathway.

**Conclusion:**

Exosomal miR-409-3p secreted from activated mast cells promotes microglial migration, activation and neuroinflammation by targeting Nr4a2 to activate the NF-κB pathway, which provides evidence that not only cytokines but also exosomal miRNAs participate in neuroinflammation. In the future, targeting exosomal miRNAs may provide new insights into neuroinflammation.

## Introduction

Neuroinflammation is a response of the central nervous system (CNS) to external stimuli, including surgery, infection and toxins, that is partly manifested by microglial activation and proinflammatory cytokine release [1]. Recently, an increasing number of studies have reported that central and peripheral mast cells play a critical role in neuroinflammation. For instance, inhibiting the neuroinflammation caused by mast cell activation can slow the progression of Parkinson’s disease [2]. Mast cells are one of the first responders that affect the neuroinflammation [3, 4]. Groot Kormelink et al. revealed that activated mast cells can release CD63-positive extracellular vesicles [5].

Exosomes, which are 30–150 nm in size, are membranous vesicles that contain mRNAs and miRNAs, are released from various cells, and are implicated in cell-to-cell communication [6]. Increasing studies have revealed that miRNAs can be derived from parent cells via exosomes and transferred into recipient cells, thereby modulating the biological characteristics of recipient cells, such as tumour metastasis or inflammatory responses [7]. miRNAs, which are one kind of noncoding RNA, decrease the expression of proteins related to biological characteristics by posttranscriptionally negatively regulating gene expression [8]. Many studies have confirmed that miRNAs participate in neuroinflammation and influence the pathogenesis of CNS diseases [9].

Moreover, in response to lipopolysaccharide (LPS) stimulation or histamine receptor triggering, mast cells can secrete cytokines and exosomes [10]. By using microarray analysis, Ekstrom [11] and Valadi [12] discovered that mast cell-derived exosomes contain miRNAs, which can be transferred to other cells and continue to function in these recipient cells. Li et al. reported that mast cells can secrete exosomal miR-223 and then deliver it to intestinal epithelial cells, resulting in the destruction of intestinal barrier function [13]. Interestingly, secretory exosomes from mast cells harbouring miRNAs are involved in communication with the nervous system [14]. In fact, microglia have been shown to be recipient cells for exogenous exosomes. Exosomes harvested from LPS-treated donor mice were infused into recipient mice and lead to enhanced neuroinflammation, and the most prominent effect was on microglia [15].

Previous studies have placed substantial emphasis on the roles of cytokines and chemokines in neuroinflammation [16]. However, to date, whether exosomal miRNAs participate in the interaction between mast cells and microglia, thus promoting neuroinflammation, has never been clearly investigated. Our previous study found evidence that products secreted from activated murine P815 cells could induce microglial activation and neuroinflammation [17]. Thus, based on this finding, the aim of this study was to show that exosomal miR-409-3p derived from LPS-stimulated murine P815 mast cells could be transferred to murine BV-2 microglial cells. Moreover, transferred miR-409-3p promoted microglial migration, activation and neuroinflammation by targeting Nr4a2 to activate the NF-κB pathway.

## Methods

### Cell culture

The source and culture method of the murine P815 mast cell line were the same as those described in our previous study [17]. LPS was obtained from Sigma-Aldrich (St. Louis, MO, USA). Murine P815 cells were stimulated with LPS (1 μg/ml) for 24 h. Then, exosomes were extracted from the cell culture supernatants. The exosomes were added to murine BV-2 cells for another 24 h. Murine BV-2 microglia were obtained from the Cell Bank of the Chinese Academy of Science (Shanghai, China). The cells were cultured in high-glucose Dulbecco’s modified Eagle’s medium (DMEM) with 10% foetal bovine serum (FBS) (Invitrogen, Carlsbad, CA) and 1% pen/strep. Cell culture experiments were performed in triplicates.

### Exosome isolation, identification and labelling

Exosomes were isolated from cell culture supernatants by ultracentrifugation. The culture supernatant samples were centrifuged at 300×*g* for 10 min, 2000×*g* for 10 min, 10,000×*g* for 30 min and 110,000×*g* at 4 °C for 70 min in succession. After washing the pellets with phosphate-buffered saline (PBS) and resuspending, the cell suspension was centrifuged again at 110,000×*g* at 4 °C for 70 min. Transmission electron microscopy (TEM, Tecnai G2 Spirit Bio TWIN, FEI, USA) was used to observe the size of the exosomes. All the isolated exosomes were fixed with glutaraldehyde (5%) and then placed into a carbon-coated copper grid that was covered with phosphotungstic acid solution (2%, pH 7.0) for 30 s. Nanoparticle-tracking analysis (NTA) was used to observe the size and distribution of the exosomes. The exosomes (10–20 mg) were dissolved in PBS (1 ml) and vortexed for 1 min. The size and distribution of the exosomes were measured by ZetaView 8.04.02 software. The exosomes were incubated with PKH67 membrane dye (4 μl, Sigma) and Diluent C (1 ml) for 4 min. The labelled exosomes were filtered by using Exoquick exosome precipitation solution, followed by suspension in basal medium. Murine BV-2 cells were incubated with the above liquid (250 μl) for 3 h and then incubated with 4% paraformaldehyde (1 ml) for half an hour. The nuclei were stained with 4′,6-diamidino-2-phenylindole (DAPI, Sigma). The images were observed by using a fluorescence microscope (Zeiss, LSM700B, Germany). Cell culture experiments were performed in triplicates.

### RT-qPCR

RNA was extracted from cells and exosomes. The method was the same as that described in our previous study [7]. The primers were as follows: miR-409-3p, forward, 5′-TGGTACTCGGAGAGAGGTTACCC-3′, and reverse, 5′-ATGGACTATCATATGCTTACCGTA-3′; IL-1β, forward, 5′-TTGACGGACCCCAAAAGAT-3′, and reverse, 5′-GAAGCTGGATGCTCTCATCTG-3′; CD86, forward, 5’-GACCGTTGTGTGTGTTCTGG-3′, and reverse, 5′-GATGAGCAGCATCCAAGGA-3′; and GAPDH, forward, 5′-AACTTTGGCATTGTGGAAGG-3′, reverse, 5′-GGATGCAGGGATGATGTTCT-3′. Cell culture experiments were performed in triplicates.

### Cell transfection

Cells were transfected with miR-409-3p mimics/mimic negative control (mimics NC) or miR-409-3p inhibitor/inhibitor negative control (inhibitor NC, GenePharma, Shanghai, China) with 8 μl Lipofectamine 3000 (Thermo Fisher Scientific, Shanghai, China). Murine BV-2 cells were transfected with miR-409-3p mimics, followed by transfection with lentiviral vectors that overexpressed Nr4a2 (Lv-Nr4a2). The empty lentiviral vector (Lv-vector) was used as the control. Cell culture experiments were performed in triplicates.

### Western blot

Proteins were extracted from cells and brain tissues and treated with RIPA lysis and extraction buffer (KeyGen Biotechnology, Nanjing, China), and then, the concentrations of these samples were measured by bicinchoninic acid (BCA) assay. The specific steps of Western blotting were the same as those described in our previous studies [17]. The antibodies were anti-CD63 (ab217345, Abcam), anti-TSG101 (ab125011, Abcam), anti-Calnexin (ab10286, Abcam), anti-Nr4a2 (ab176184, Abcam), anti-NF-κB p65 (ab16502, Abcam) and anti-GAPDH (ab9485, Abcam). Cell culture experiments were performed in triplicates.

### Transwell assay

Using chamber inserts in a Transwell apparatus (Millipore, MA, USA), murine BV-2 cells (2 × 10^4^) were resuspended in DMEM, plated in the upper chamber and treated with isolated exosomes or mimics NC/mimics. DMEM (600 μl) were added into the lower chamber. The cells were incubated for 24 h at 37 °C, fixed in 4% paraformaldehyde for half an hour, and stained with 0.2% crystal violet for an hour. The images were obtained by using NIS Elements software (Nikon, Tokyo, Japan). Cell culture experiments were performed in triplicates.

### ELISA

ELISA kits (R&D Systems) were used to detect the amounts of TNF-α and IL-6 in the cell culture supernatants. Cell culture experiments were performed in triplicates.

### Immunofluorescence staining

Iba1 and CD68 were used to evaluate murine BV-2 microglial activation. Cells or brain tissues were fixed with 4% paraformaldehyde for half an hour. Nonspecific binding was blocked by incubating the cells in 5% BSA (0.1% Triton X-100) for an hour. The slides were incubated with Iba1 antibody (GB11105, 1:500 dilution), CD68 antibody (GB11067, 1:1000 dilution) and horseradish peroxidase (HRP)-conjugated goat anti-rabbit IgG (H+L) (GB23303, 1:500 dilution) (Servicebio Technology Co. Ltd., Wuhan, China) and then incubated with fluorescein isothiocyanate (FITC) or Cy3. In cell culture experiments, Iba1 (FITC, green), CD68 (Cy3, red). Cell culture experiments were performed in triplicates. In vivo experiments, Iba1 (Cy3, red), CD68 (FITC, green). After 10 min, the slides were washed with Tris-buffered saline tween (TBST) 3 times for 5 min each. The nuclei were stained with 4′6-diamidino-2-phenylindole (DAPI) and incubated for 10 min. The images were observed by using a confocal microscope.

### Luciferase

The 3′-UTRs of the Nr4a2 wild-type (WT) or mutant (Mut) binding sequences were synthesized by GeneScript (Nanjing, China). Murine BV-2 cells transfected with miR-409-3p mimics or mimics NC were seeded into 96-well plates and co-transfected with Nr4a2-WT or Nr4a2-Mut. The firefly and Renilla luciferase values were measured by using a Dual-Luciferase® Assay Kit (Promega, Madison, WI, USA). Cell culture experiments were performed in triplicates.

### In vivo experiment

Mice (male, 6–10 weeks old) were purchased from the Model Animal Research Center of Nanjing University (China) and were housed at 22.0 ± 1.0 °C and 40% humidity. Our animal experiments were approved by the Nanjing Medical University Animal Care and Use Committee. Murine P815-miR-409-3p-mimics NC-exosomes (mimics NC exo) and murine P815-miR-409-3p mimics-exosomes (mimics exo) (200 μg exosomes precipitated in 200 μl PBS per mouse for each day) were administered to C57BL/6 mice by tail vein injection for three days. The same volume of PBS was administered to the control group. The mice were anaesthetized (2.1% isoflurane anaesthesia) and sacrificed, and then, brain tissues were obtained for immunofluorescence and Western blotting.

### Statistical analysis

The statistical analysis was performed by using STAT11 and GraphPad software 8.0. This study conducted Student’s *t* test for two-group comparisons and one-way or two-way ANOVA for more than two-group comparisons. The data are presented as the mean ± SEM. *P* < 0.05 was considered statistically significant.

## Results

### Exosomes secreted from activated mast cells promoted microglial migration, activation and neuroinflammation

**Fig. 1:**
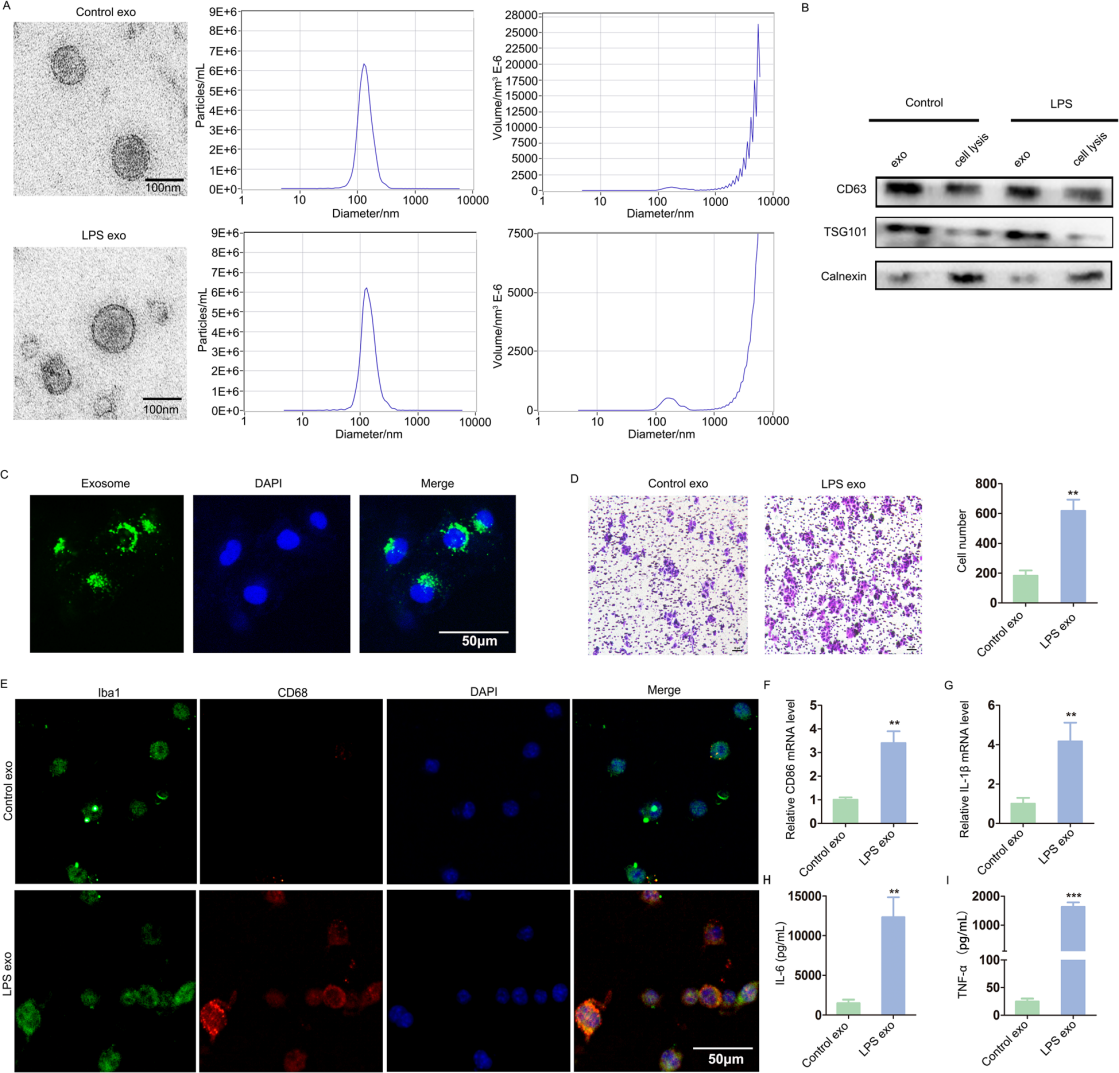
Exosomes secreted from activated mast cells promoted microglial migration, activation and neuroinflammation. **a** Exosomes were extracted from the cell culture supernatants of murine P815 cells (Control exo) or LPS-P815 cells (LPS exo). The shapes and size distributions of these exosomes were observed through TEM and NTA. Scale bar 100 nm. **b** Representative exosomal markers were detected by Western blot. **c** Fluorescence microscopy showed the uptake of PKH67-labelled exosomes by murine BV-2 cells. Scale bar 50 μm. **d** Murine BV-2 microglial migration was evaluated through Transwell assays. Scale bar 10 μm. E, Immunofluorescence staining for Iba1 and CD68 was used to evaluate microglial activation. Scale bar 50 μm. F-I, CD86, IL-1β and inflammatory cytokines (IL-6 and TNF-α) were detected via qRT-PCR and ELISA. Cell culture experiments were performed in triplicates. *P < 0.05; **P < 0.01; ***P < 0.001

First, exosomes were extracted from murine P815 cell culture supernatants, and their shapes were observed by TEM. The exosomes were 40–150-nm-diameter vesicles with a uniform cup-shaped morphology, and the size distribution was measured by NTA (Fig. 1a). To further determine whether the collected vesicles were exosomes, representative exosome markers were detected by western blot. As shown in Fig. 1b, increased CD63 and TSG101 expression and decreased calnexin expression were observed in the isolated exosomal fraction. Next, murine BV-2 cells were cultured with PKH67-labelled exosomes. Fluorescence microscopy showed that the exosomes were efficiently taken up by murine BV-2 cells (Fig. 1c).

Microglial migration is one of the pathological characteristics of the CNS after injury. Migrating activated microglia mediate inflammation through phagocytosis, antigen presentation and factor secretion [18, 19]. To investigate the effect of exosomes on murine BV-2 microglial migration, we performed a Transwell assay. As shown in Fig. 1d, the number of migrating cells in the LPS-P815 exosomes group (LPS exo) was greater than that in the control group (Control exo), suggesting that LPS-P815 exosomes accelerated murine BV-2 microglial migration.

Immunofluorescence staining for Iba1 and CD68 was used to evaluate microglial activation. Significantly, LPS-P815 exosomes induced murine BV-2 microglial activation (Fig. 1e). To further explore the effect of LPS-P815 exosomes on murine BV-2 microglial activation, CD86, IL-1β, IL-6 and TNF-α were detected. Notably, LPS-P815 exosomes led to increased levels of CD86 and IL-1β (Fig. 1f, g). The secretion of IL-6 and TNF-α was remarkably increased in the LPS-P815 exosomes group (Fig. 1h, i). These results revealed that LPS-P815 exosomes promoted microglial activation and neuroinflammation.

### Activated mast cells delivered exosomal miR-409-3p to murine BV-2 microglia

**Fig. 2:**
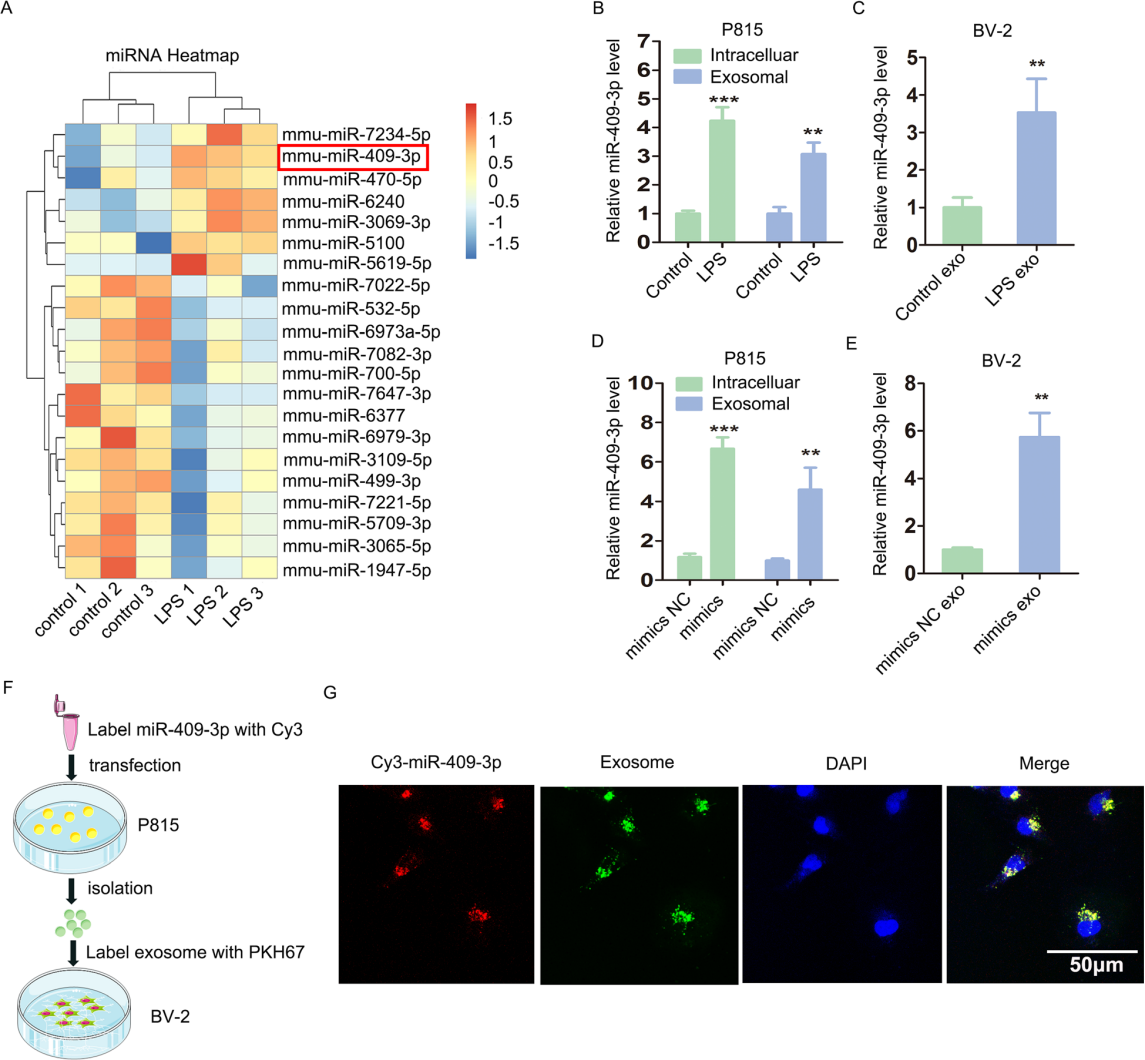
Activated mast cells delivered exosomal miR-409-3p to murine BV-2 microglial cells. **a** Microarray analysis (*P* < 0.05, FC > 1.5) indicated that miR-409-3p was increased in LPS-P815 exosomes (LPS exo: LPS1, LPS2, LPS3; Control exo: control1, control2, control3). **b** Confirmation by qRT-PCR showed that miR-409-3p levels were increased in LPS-P815 cells and in the corresponding exosomes. **c** Murine BV-2 cells were incubated with control exosomes (Control exo) or LPS-P815 exosomes (LPS exo). The miR-409-3p levels in the two groups were detected by qRT-PCR. **d** Murine P815 cells were transfected with miR-409-3p mimics or mimics NC. The miR-409-3p levels in the cells and exosomes were detected by qRT-PCR. **e** Murine BV-2 cells were incubated with P815-miR-409-3p mimics NC-exosomes group (mimics NC exo) or P815-miR-409-3p mimics-exosomes group (mimics exo). The miR-409-3p levels were detected by qRT-PCR. F, Murine P815 cells were transfected with Cy3-labelled miR-409-3p, and exosomes were isolated from murine P815 cell culture supernatants. The isolated exosomes were labelled with PKH67 and then incubated with murine BV-2 cells. G, Fluorescence microscopy revealed that miR-409-3p could be efficiently taken up by murine BV-2 cells via exosomes. Scale bar 50 μm. Cell culture experiments were performed in triplicates. *P < 0.05; **P < 0.01; ***P < 0.001

Using microarray analysis, we found that compared with the control group, in the LPS-treated group, 7 exosomal miRNAs (mmu-miR-6240, mmu-miR-3069-3p, mmu-miR-5100, mmu-miR-7234-5p, mmu-miR-470-5p, mmu-miR-5619-5p and mmu-miR-409-3p) were upregulated, and 14 exosomal miRNAs (mmu-miR-7647-3p, mmu-miR-6979-3p, mmu-miR-7022-5p, mmu-miR-6973a-5p, mmu-miR-3065-5p, mmu-miR-5709-3p, mmu-miR-7082-3p, mmu-miR-7221-5p, mmu-miR-532-5p, mmu-miR-6377, mmu-miR-1947-5p, mmu-miR-700-5p, mmu-miR-3109-5p and mmu-miR-499-3p) were downregulated (*P* < 0.05, FC > 1.5, Fig. 2a). Furthermore, through qRT-PCR confirmation, we found that miR-409-3p was increased in LPS-P815 cells and in the corresponding exosomes (Fig. 2b). Then, we also found that miR-409-3p was increased in murine BV-2 cells incubated with LPS-P815 exosomes (LPS exo) (Fig. 2c).

Moreover, as shown in Fig. 2d, miR-409-3p was increased 7-fold and exosomal miR-409-3p was increased 4-fold in the mimic group relative to the mimic NC group. Moreover, an increased level of miR-409-3p was observed in the murine BV-2 cells cultured with P815-miR-409-3p mimics exosomes (mimics exo) (Fig. 2e). Additionally, murine P815 cells were transfected with Cy3-labelled miR-409-3p, and exosomes were isolated from the murine P815 cell culture supernatants. The isolated exosomes were labelled with PKH67 and then incubated with murine BV-2 cells (Fig. 2f). Fluorescence microscopy revealed that miR-409-3p could be efficiently taken up by murine BV-2 cells via exosomes (Fig. 2g).

### Upregulated miR-409-3p promoted microglial migration, activation and neuroinflammation

**Fig. 3:**
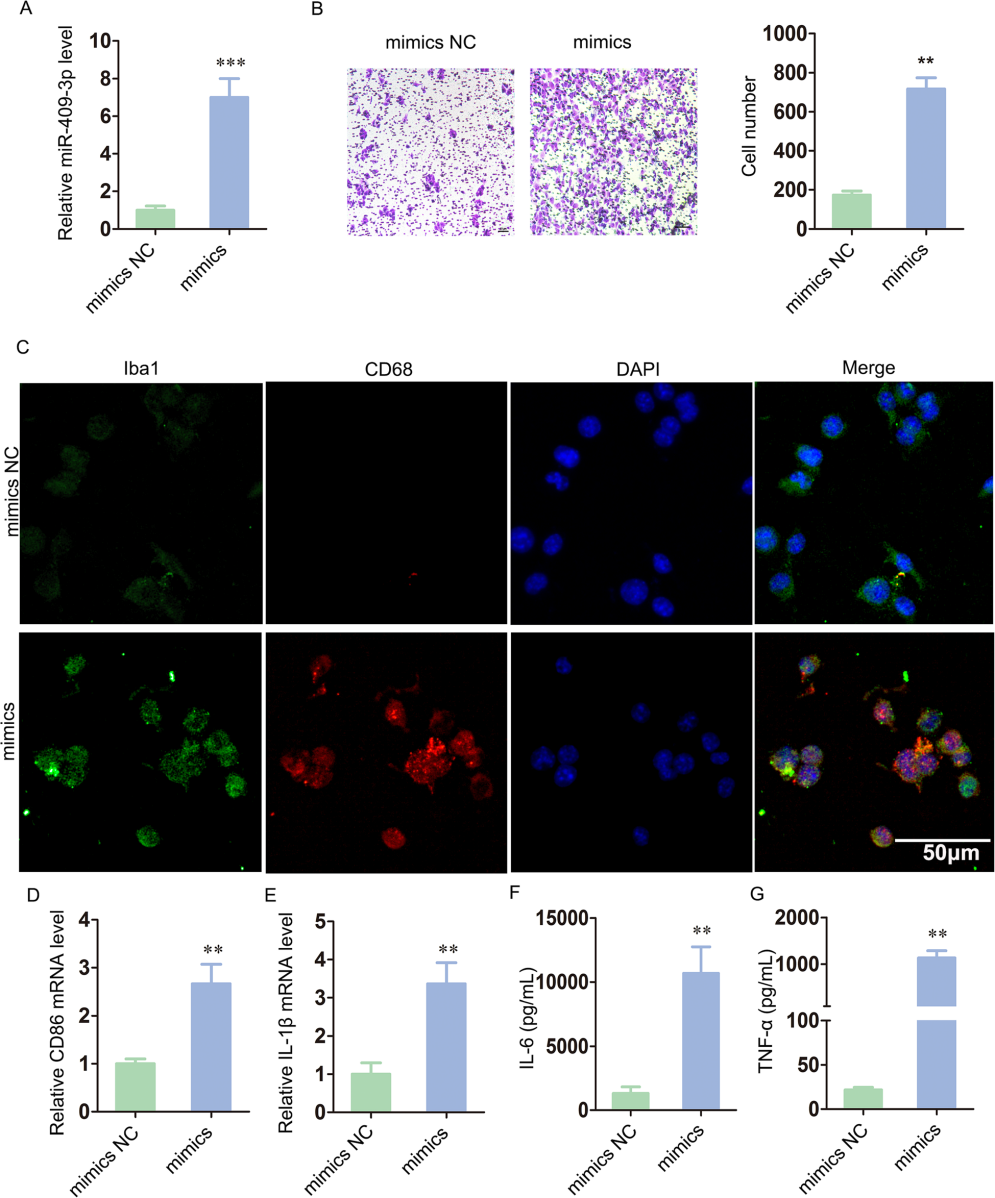
MiR-409-3p promoted murine BV-2 microglial migration, activation and neuroinflammation. **a** Murine BV-2 cells were transfected with miR-409-3p mimics NC or miR-409-3p mimics. The miR-409-3p levels were detected by qRT-PCR. **b** The migratory ability of murine BV-2 cells was measured by Transwell assay. Scale bar 10 μm. **c** Immunofluorescence staining for Iba1 and CD68 was used to evaluate murine BV-2 microglial activation. Scale bar 50 μm. **d**–**g** CD86, IL-1β and inflammatory cytokines (IL-6, TNF-α) were detected via qRT-PCR and ELISA. Cell culture experiments were performed in triplicates. *P < 0.05; **P < 0.01; ***P < 0.001

To make the effect of increased miR-409-3p on murine BV-2 cells more significant, we transfected miR-409-3p mimics into murine BV-2 cells to simulate the delivery of exosomal miR-409-3p secreted from activated murine P815 cells (Fig. 3a). Transwell assays revealed an increased number of migrating cells in the miR-409-3p mimic group relative to that in the mimic NC group (Fig. 3b).

As shown in Fig. 3c, miR-409-3p could induce murine BV-2 microglial activation. As expected, miR-409-3p increased the expression of CD86 and IL-1β (Fig. 3d, e). Moreover, the production of proinflammatory cytokines (IL-6 and TNF-α) was also increased in the mimic group (Fig. 3f, g). In summary, these findings demonstrated that miR-409-3p promoted microglial migration, activation and neuroinflammation.

### miR-409-3p acted as a pro-neuroinflammatory molecule by targeting Nr4a2 to activate the NF-κB pathway

**Fig. 4:**
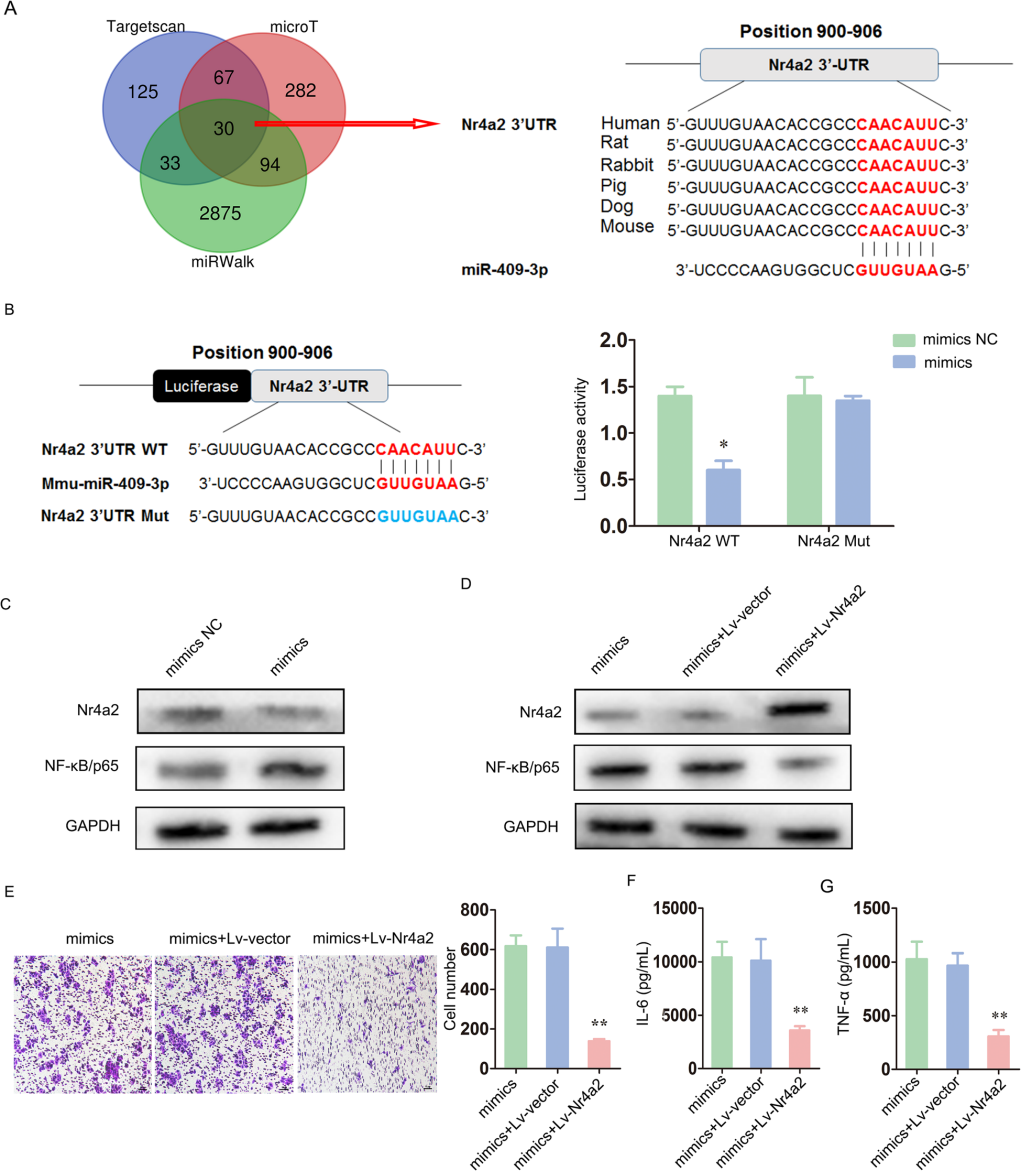
miR-409-3p acted as a pro-neuroinflammatory molecule by targeting Nr4a2 to activate the NF-κB pathway. **a** The intersecting outputs from three prediction software programs (TargetScan, miRWalk and microT) included 30 potential target genes. Of these genes, the 3′-UTR of Nr4a2 bound to miR-409-3p with a high score and high conservation. **b** 3′-UTRs with Nr4a2 wild-type (WT) or mutant (Mut) binding sequences were synthesized. The luciferase reporter assay indicated that Nr4a2 was a direct target of miR-409-3p. **c** The protein expression levels of Nr4a2 and NF-κB/p65 were detected by western blot. **d** Murine BV-2 cells were co-transfected with miR-409-3p mimics and Lv-Nr4a2 (mimics + Lv-Nr4a2). The transfection efficiency and corresponding changes in NF-κB/p65 expression were also detected by western blot. E, The migratory ability of murine BV-2 cells was measured by Transwell assay. Scale bar 10 μm. F-G, The IL-6 and TNF-α levels were investigated by ELISA. Cell culture experiments were performed in triplicates. *P < 0.05; **P < 0.01; ***P < 0.001

To identify the candidate target genes of miR-409-3p, we collected and intersected the outputs from three prediction software programs (TargetScan, miRWalk and microT). Of the 30 intersecting genes, the 3′-UTR of Nr4a2 bound to miR-409-3p with a high score and high conservation (Fig. 4a). Importantly, nuclear receptor subfamily 4 group A member 2 (Nr4a2), also known as nuclear receptor-related 1 protein (Nurr1), is strongly expressed in the hippocampus, substantia nigra, temporal cortex and subiculum [20, 21]. Nr4a2 contributes to cognitive functions by mediating hippocampal neurogenesis and is a therapeutic target for the treatment of neuroinflammation [22, 23]. Briana et al. identified Nr4a2 as a suppressor of the nuclear factor kB (NF-κB) pathway in murine BV-2 microglia [24].

Thus, via a luciferase reporter assay, we found decreased luciferase intensity in the miR-409-3p mimic and Nr4a2-WT groups relative to that in the control groups, which indicated that Nr4a2 was a direct target of miR-409-3p (Fig. 4b). Moreover, Western blot analysis showed that high miR-409-3p levels downregulated Nr4a2 protein expression and upregulated NF-κB protein expression (Fig. 4c).

Furthermore, we co-transfected miR-409-3p mimics and Lv-Nr4a2 into murine BV-2 cells to assess whether the regulatory effect of miR-409-3p depended on Nr4a2. The transfection efficiency and corresponding changes in NF-κB/p65 expression are shown in Fig. 4d. Transwell assays showed that the number of migrating cells in the miR-409-3p mimics and Lv-Nr4a2 groups (mimics + Lv-Nr4a2) was partly reduced compared with that in the control groups (Fig. 4e). Similarly, the IL-6 and TNF-α levels were clearly attenuated (Fig. 4f, g).

Overall, these data support the view that miR-409-3p acted as a pro-neuroinflammatory molecule by targeting Nr4a2 to activate the NF-κB pathway.

Exosome-mediated transfer of miR-409-3p promoted microglial migration, activation and neuroinflammation.

**Fig. 5:**
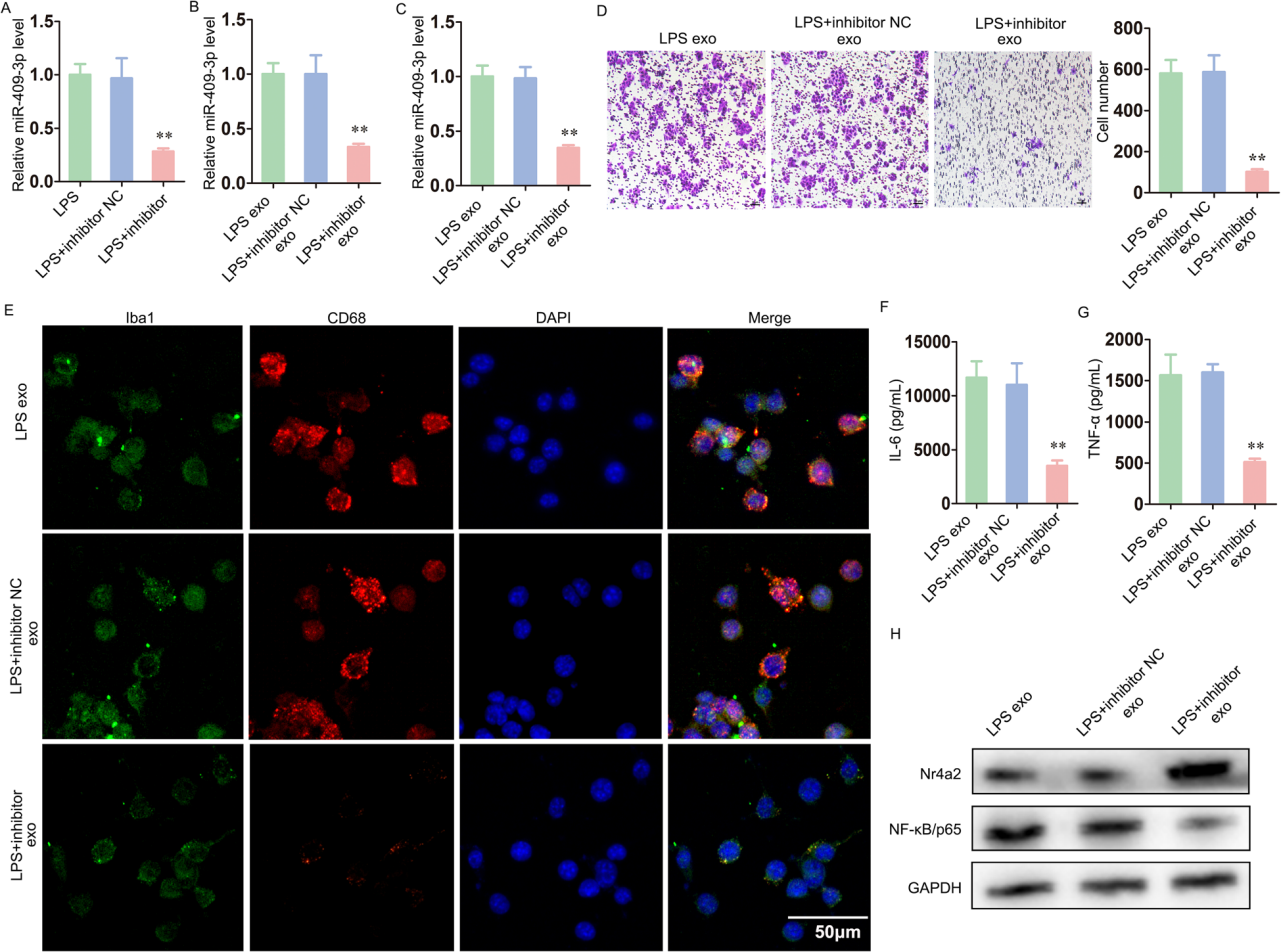
Exosome-mediated transfer of miR-409-3p promoted microglial migration, activation and neuroinflammation. **a**, **b** After murine P815 cells were transfected with miR-409-3p inhibitor or inhibitor NC, all the cells were treated with LPS. The relative miR-409-3p levels of both the cells and exosomes were determined via qRT-PCR. **c** Murine BV-2 cells were cultured with the corresponding exosomes extracted from these three groups (LPS exo, LPS + inhibitor NC exo, LPS + inhibitor exo). The relative miR-409-3p levels in murine BV-2 cells were detected via qRT-PCR. **d** The migration of murine BV-2 microglia was tested by Transwell assay. Scale bar 10 μm. **e** Immunofluorescence staining for Iba1 and CD68 was used to evaluate murine BV-2 microglial activation. Scale bar 50 μm. **f**, **g** The IL-6 and TNF-α levels were investigated by ELISA. H, The protein expression levels of Nr4a2 and NF-κB/p65 were detected by Western blot. Cell culture experiments were performed in triplicates. *P < 0.05; **P < 0.01; ***P < 0.001

To further confirm the role of the exosome-mediated transport of miR-409-3p to murine BV-2 microglia, we performed a rescue experiment. After murine P815 cells were transfected with a miR-409-3p inhibitor or inhibitor NC, all the cells were treated with LPS. Compared to the control groups, the murine P815 cells transfected with a miR-409-3p inhibitor and treated with LPS exhibited reduced miR-409-3p levels in both the cells and exosomes (Fig. 5a, b). Then, murine BV-2 cells were cultured with the corresponding exosomes extracted from these three groups. Similar to the results described above, the relative miR-409-3p level was reduced in the murine BV-2 cells cultured with LPS + inhibitor exosomes (LPS + inhibitor exo) (Fig. 5c). As shown in Fig. 5d, e, both the migration and activation of murine BV-2 microglia were decreased in the LPS + inhibitor exosomes group compared with the control groups. The IL-6 and TNF-α levels were also clearly decreased in the LPS + inhibitor exosomes group (Fig. 5f, g). In addition, Western blot analysis showed upregulated Nr4a2 and downregulated NF-κB protein levels in the LPS + inhibitor exosomes group (Fig. 5h).

### Exosomes secreted by mast cells rich in miR-409-3p promoted microglial activation

**Fig. 6:**
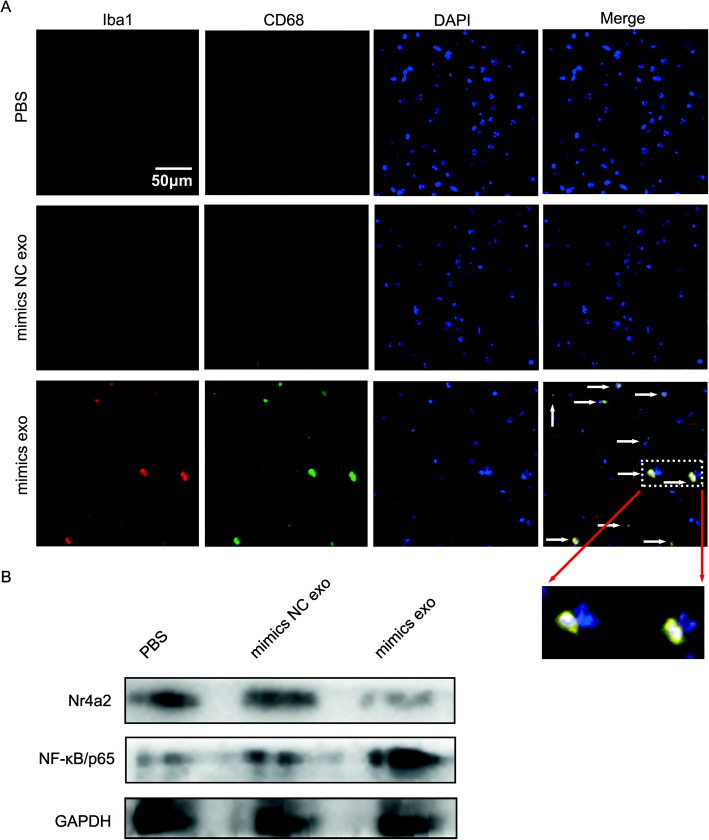
Exosomes secreted by mast cells rich in miR-409-3p promoted microglial activation. **a** Iba1 and CD68 was used to detect microglial activation. Immunofluorescence assays were used to measure the Iba1 and CD68 levels in the hippocampus in the PBS group, P815-miR-409-3p mimics NC-exosomes group (mimics NC exo) and P815-miR-409-3p mimics-exosomes group (mimics exo). Scale bar 50 μm. **b** Western blotting was used to detect Nr4a2 and NF-κB/p65 protein expression. *n* = 5 C57BL/6 mice per group

Finally, in vivo, PBS, P815-miR-409-3p mimics NC-exosomes (mimics NC exo) and P815-miR-409-3p mimics-exosomes (mimics exo) were administered to C57BL/6 mice by tail vein injection. Iba1 was used to detect microglia, and CD68 was used to detect microglial activation. After 3 days, through immunofluorescence assay, we found that compared with the PBS group and mimic NC exo group, the mimics exo group exhibited increased microglial activation (Fig. 6a). Moreover, Western blot analysis showed that decreased Nr4a2 and increased NF-κB/p65 levels were observed in the mimics exo group (Fig. 6b). These results initially demonstrated that mast cell-derived miR-409-3p could cause microglial activation.

## Discussion

**Fig. 7:**
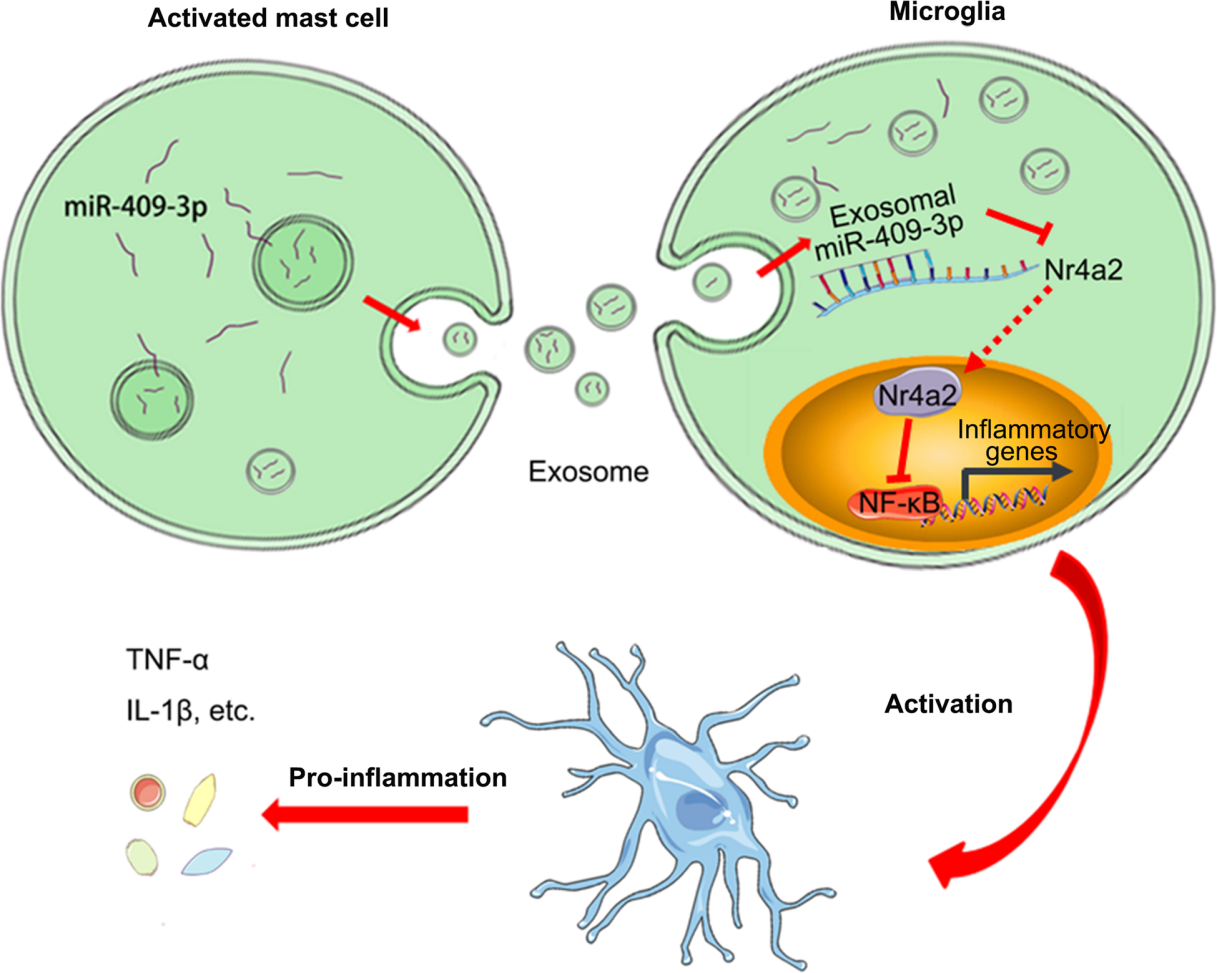
The concept of our study was summarized via a pattern diagram

Prior studies have placed substantial emphasis on the roles of cytokines and chemokines in the functional aspects of mast cell-microglia interactions during neuroinflammation [25]. Importantly, we demonstrated a new regulatory mechanism that occurs via exosome-mediated cell-to-cell communication; our findings suggested that exosomal miRNAs participate in the interaction between mast cells and microglia and thus promote neuroinflammation. As shown in the pattern diagram in Fig. 7, activated mast cells transfer exosomal miR-409-3p to microglia, downregulate Nr4a2 expression and activate the NF-κB pathway, thereby promoting microglial migration, activation and neuroinflammation.

Our previous study showed that the cell culture supernatants of LPS-activated P815 cells could induce microglial activation and neuroinflammation [17], which led us to question whether this effect might be partly caused by other products released from LPS-activated P815 cells. Mast cells are known to be sources of exosomes that contribute to inflammation [26]. Thus, we obtained high numbers exosomes from LPS-stimulated P815 cells, which was consistent with other research [12]. Microglial activation has a dual effect on neuroinflammation. On the one hand, M1 polarisation exerts a harmful effect by releasing proinflammatory cytokines (TNF-α, IL-6 and IL-1β). On the other hand, M2 polarisation exerts a beneficial effect by secreting anti-inflammatory cytokines (TGF-β, IL-10 and IL-4) [27, 28]. Interestingly, exosomes were efficiently taken up by murine BV-2 microglia and led to increases in the levels of CD86, IL-1β and inflammatory cytokines (IL-6, TNF-α), which suggested that LPS-P815 exosomes promoted microglial activation and neuroinflammation. An in vivo experiment revealed that after exposure to LPS, the ratio of exosome uptake by recipient mouse microglia was 86.8%, and many inflammation-related microRNAs (miR-15a, miR-15b, miR-21, miR-27b, miR-125a, miR-146a and miR-155) were observed at significantly higher levels in these exosomes [15]. This in vivo experiment also confirmed that exosomal miRNAs might pass through the brain-blood barrier (BBB) by impairing tight junctions and enhancing BBB permeability, which provided new ideas for our future research.

Importantly, our study was the first to reveal the following: (1) exosomes secreted from activated mast cells promoted microglial migration, activation and neuroinflammation; (2) miR-409-3p levels were increased in activated mast cells and exosomes and were transferred to murine BV-2 microglia via exosomes; (3) upregulated miR-409-3p promoted microglial migration, activation and neuroinflammation by targeting Nr4a2 to activate the NF-κB pathway; and (4) exosome-mediated transfer of miR-409-3p promoted microglial migration, activation and neuroinflammation.

Emerging studies have demonstrated that miRNAs promote microglial activation by modulating target genes [29]. Through microarray analysis, we found increased levels of miR-409-3p in LPS-P815 cells and in the corresponding exosomes. Moreover, miR-409-3p levels were increased in murine BV-2 cells incubated with LPS-P815 exosomes. Liu et al. reported that miR-409-3p is implicated in the IL-17-induced release of inflammatory cytokines by astrocytes and in the pathogenesis of experimental autoimmune encephalomyelitis in mice through the regulation of the SOCS3/STAT3 pathway [30]. Here, we found that upregulated miR-409-3p levels promoted murine BV-2 microglial migration, activation and neuroinflammation. Based on the intersecting outputs from the three prediction software programs, we focused on the target gene Nr4a2 to further explore the molecular mechanism by which miR-409-3p affected murine BV-2 microglia. A recent study on Nr4a2 was consistent with the evidence that Nr4a2 might exert its neuroprotective effects via its function in neurons [31]. By using a luciferase reporter assay and Western blotting, our work indicated a negative regulatory mechanism between miR-409-3p and Nr4a2, which led to activation of the NF-κB pathway.

However, there are several limitations of this study. (1) Exosomes also contain inflammatory factors and other molecules that affect inflammation. This study only discussed the effect of exosomal miRNAs on inflammation. The inflammatory response is a complex process in which multiple molecules and pathways interact and regulate each other. Therefore, the specific mechanism of the inflammatory response still needs to be further explored. (2) This study mainly performed in vitro experiments. In addition, the in vivo experimental design was relatively simple, and those experiments were only preliminary studies. Thus, further in-depth studies will be carried out with clinical specimens and animal models in the future. Regardless, our study was the first to reveal that exosomal miRNAs are involved in the mast cell-microglia interactions related to neuroinflammation.

## Conclusion

In summary, our study showed that exosomal miR-409-3p secreted from activated mast cells promotes microglial migration, activation and neuroinflammation by targeting Nr4a2 to activate the NF-κB pathway. These findings provide evidence that not only cytokines or chemokines but also exosomal miRNAs participate in the progression of neuroinflammation. In the future, targeting exosomal miRNAs may provide new insights for the treatment of neuroinflammation.

## Data Availability

The data generated during this study are available from the corresponding author on reasonable request.

## Abbreviations

TEM: Transmission electron microscopy
NTA: Nanoparticle-tracking analysis
CNS: Central nervous system
LPS: Lipopolysaccharide
BBB: Brain-blood barrier
DAPI: 4′6-Diamidino-2-phenylindole
FITC: Fluorescein isothiocyanate
